# Gene expression atlas of pigeonpea and its application to gain insights into genes associated with pollen fertility implicated in seed formation

**DOI:** 10.1093/jxb/erx010

**Published:** 2017-02-23

**Authors:** Lekha T. Pazhamala, Shilp Purohit, Rachit K. Saxena, Vanika Garg, L. Krishnamurthy, Jerome Verdier, Rajeev K. Varshney

**Affiliations:** 1 International Crops Research Institute for the Semi-Arid Tropics (ICRISAT), Hyderabad 502 324, India; 2 INRA – Research Institute in Horticulture and Seeds (IRHS), 49071 Beaucouze, France; 3 School of Plant Biology and Institute of Agriculture, University of Western Australia, 35 Stirling Highway, Crawley, WA, 6009, Australia

**Keywords:** *Cajanus cajan*, CcGEA, gene clustering, gene expression atlas, gene networking, legume genomics, male sterile genotype, pigeonpea, pollen-specific SF3, sucrose–proton symporter.

## Abstract

Pigeonpea (*Cajanus cajan*) is an important grain legume of the semi-arid tropics, mainly used for its protein rich seeds. To link the genome sequence information with agronomic traits resulting from specific developmental processes, a *Cajanus cajan* gene expression atlas (CcGEA) was developed using the Asha genotype. Thirty tissues/organs representing developmental stages from germination to senescence were used to generate 590.84 million paired-end RNA-Seq data. The CcGEA revealed a compendium of 28 793 genes with differential, specific, spatio-temporal and constitutive expression during various stages of development in different tissues. As an example to demonstrate the application of the CcGEA, a network of 28 flower-related genes analysed for *cis*-regulatory elements and splicing variants has been identified. In addition, expression analysis of these candidate genes in male sterile and male fertile genotypes suggested their critical role in normal pollen development leading to seed formation. Gene network analysis also identified two regulatory genes, a pollen-specific SF3 and a sucrose–proton symporter, that could have implications for improvement of agronomic traits such as seed production and yield. In conclusion, the CcGEA provides a valuable resource for pigeonpea to identify candidate genes involved in specific developmental processes and to understand the well-orchestrated growth and developmental process in this resilient crop.

## Highlight

A gene expression atlas of pigeonpea revealed spatio-temporal gene expression, co-expressed gene clusters and an important gene network critical for normal pollen and seed development.

## Introduction

Pigeonpea is an important, resilient crop of the semi-arid tropics, well-suited to the dryland cropping system. It is a cross-pollinated diploid (2*n*=2*x*=22) food legume with an estimated genome size of 833.07 Mb (Varshney *et al.*, 2012). It is mostly grown in marginal environments with low inputs and poor management practices in Asia, Africa and parts of Central America. In these developing countries, it is a major source of protein (23–30% seed protein content) and therefore plays a vital role in alleviating malnutrition, especially in children. Although it is a key staple food crop in these regions, limited efforts have been made to enhance its productivity, which has remained less than 1 ton ha^–1^ for over six decades. Pigeonpea productivity has been greatly challenged by various biotic and abiotic stresses. Recently, a cytoplasmic genetic male sterility based hybrid system has been established in this crop and has demonstrated improved productivity, breaking the long-standing yield barrier (Saxena *et al.*, 2010). Pigeonpea has gained global attention due to continuously increasing demand in the developing world and lack of the desired amount of seeds in the international market.

Genomics approaches have proved to be efficient in overcoming production constraints in a number of crop species (Varshney *et al.*, 2013; Kole *et al.*, 2015). In the case of pigeonpea, availability of the draft genome sequence (Varshney *et al.*, 2012) and a range of genomic resources have provided an opportunity to undertake genomics-assisted breeding (GAB). These genomic resources include the genome sequence, molecular markers, genetic maps, quantitative trait loci (QTLs), and transcriptome assembly (Pazhamala *et al.*, 2015). Furthermore in pigeonpea, expression studies using transcriptome sequencing and quantitative real-time PCR have been conducted to understand the plant’s response to abiotic stresses (drought and salinity; Sinha *et al.*, 2015) and biotic stresses (fusarium wilt and sterility mosaic disease; Singh *et al.*, 2016), and to study pod and seed development (Pazhamala *et al.*, 2016). However, a baseline study of all the tissues from different developmental stages such as a gene expression atlas to increase the efficiency of such approaches has been lacking in pigeonpea. A gene expression atlas has been developed in several crops especially to define subsets of genes expressed in different tissue systems using either DNA microarray or RNA-seq approaches. In legumes, gene expression atlases are available for *Medicago truncatula* (Benedito *et al.*, 2008), *Lotus japonicus* (Verdier *et al.*, 2013), *Glycine max* (Libault *et al.*, 2010; Severin *et al.*, 2010), *Phaseolus vulgaris* (O’Rourke *et al.*, 2014), *Pisum sativum* (Alves-Carvalho *et al.*, 2015), *Vigna unguiculata* (Yao *et al.*, 2016) and *Arachis hypogaea* (Clevenger *et al.*, 2016). These studies aimed at investigating the complex biological processes underlying pod development, seed maturation, nodulation, and symbiosis.

The advent of the next generation sequencing technology has made sequencing of many non-model crops feasible in recent years. Pigeonpea is one of the few crops for which the technology was adopted early on to develop a draft genome sequence using the ‘Asha’ genotype. The genome sequence of pigeonpea has provided useful insights into the protein coding regions and gene functions, and clues to biological processes. However, this information was mainly based on the homology and *de novo* gene prediction programs. In order to correlate and complement the genome information with the gene expression that is modulated in a temporal and spatial manner, a gene expression atlas with 30 samples (tissues) collected from the ‘Asha’ (ICPL 87119) genotype has been developed. The Asha pigeonpea genotype is a widely cultivated, high yielding, medium duration inbred line resistant to several important diseases (fusarium wilt and sterility mosaic disease), for which a number of genetic and genomic resources including a draft genome have been developed. In the present study, a gene expression atlas has been developed for pigeonpea that reports the identification and quantification of genes exhibiting spatio-temporal expression using 30 diverse tissues. Further, an example has been provided to elucidate the efficacy of this comprehensive dataset to identify a co-expressed gene network exclusive to floral tissues (bud, flower, stamen, pistil, sepal and petal). The targeted tissues are highly specialized and their development is tightly controlled and coordinated for effective fertilization and production of viable seeds. The candidate genes identified were associated to pollen fertility and seed setting, which have specific implications for the key agronomic trait of yield for their possible deployment in GAB in pigeonpea.

## Materials and methods

### Plant material

Seeds of the ‘Asha’ genotype (ICPL 87119) were sown in three different sets under glasshouse conditions by maintaining 26 °C day/22 °C night temperature with a photoperiod of 13 h day/11 h night. These sets included seeds germinated in (i) Petri plates with filter paper, (ii) paper cups containing sterile sand, and (iii) pots containing sterile black soil and sand (1:1). All these three sets of experiments were set up in three biological replicates. Set (i) was used for harvesting tissues from the germinal stage, set (ii) for the seedling stage, and set (iii) for the vegetative, reproductive and senescence stages (Supplementary Fig. S1 at *JXB* online). Tissues from the germinal stages (embryo, hypocotyl, radicle, and cotyledons) and other flower/seed tissues (stamen, pistil, petal, sepal, and immature seeds) were carefully dissected on ice and immediately frozen in liquid nitrogen. Root tissues and nodules from all the stages were excised after brief washes in diethyl pyrocarbonate-treated water followed by flash freezing in liquid nitrogen. All other aerial tissues including leaves, stem, petioles, pods, shoot apical meristem, flowers and buds were excised from plants and directly frozen in liquid nitrogen. All the tissues were stored at −80 °C until total RNA isolation.

### RNA extraction, Illumina sequencing and data pre-processing

Total RNA was isolated using the Nucleospin RNA plant kit (Macherey-Nagel, Duren, Germany) as per the manufacturer’s instructions. The qualitative and quantitative assessments of these total RNA samples were conducted using an Agilent 2100 Bioanalyzer (Agilent Technologies, Palo Alto, CA, USA). RNA samples with RNA integrity (RIN) value ≥8 were pooled in equimolar amounts from three biological replicates prior to library preparation and subsequent sequencing. The cDNA libraries were prepared using an Illumina TruSeq RNA Sample Preparation Kit (Illumina Inc., San Diego, CA, USA) following the manufacturer’s instructions. Pair-end sequencing was performed in two sets: a set of 20 samples (nos 1–20) was sequenced using an Illumina HiSeq 2000 at Genotypic Technology Pvt. Ltd, India and a second set of 10 samples (nos 21–30) was sequenced in-house using an Illumina HiSeq 2500 sequencing system. The raw sequencing data were subjected to quality check to ensure high quality reads for downstream analyses. Reads with Phred score <20, read length <50 bases, and consisting of any uncalled bases using NGSQC Box (Katta *et al.*, 2015) were filtered out.

### Data analysis

An open source software pipeline, Tuxedo suite (Trapnell *et al.*, 2012*a*), was used for analysing the RNA-seq data (deposited in NCBI Sequence Read Archive (SRA) database with BioProject ID PRJNA354681). The reads were mapped on the pigeonpea genome (Varshney *et al.*, 2012; http://www.icrisat.org/gt-bt/iipg/genomedata.zip) using a splice-aware alignment algorithm, TopHat (v 2.1.0) (Kim *et al.*, 2013). Thereafter, the mapped reads were assembled into transcripts and their abundances were estimated using Cufflinks (v 2.1.1) (Trapnell *et al.*, 2010).

#### Identification of differentially expressed genes

The differentially expressed genes (DEGs) with log_2_ fold change values ≥2 and ≤–2 (respectively up-regulated and down-regulated) and *P*-value ≤0.05 were identified using Cuffdiff (Trapnell *et al.*, 2012*b*) displaying significance level as ‘Yes’. CummeRbund (Trapnell *et al.*, 2012*b*), a part of the Tuxedo suite (http://compbio.mit.edu/cummeRbund/index.htmland), was used to visualize the differential gene expressions between tissues as scatter plots, also called volcano plots. The identified DEGs were annotated using Blast2GO v 3.3 (Conesa *et al.*, 2005) against the NCBI non-redundant (nr) Viridiplantae protein database.

#### Co-expressed gene network analysis

The co-expressed gene modules were identified and a topological overlap matrix (TOM) plot was generated using Weighted Gene Co-expressed Network Analysis (WGCNA; Langfelder and Horvath, 2007, 2008). The identified gene modules were further visualized using Cytoscape v 3 (Lopes *et al.*, 2010). In addition, the PlantCARE (Lescot *et al.*, 2002) database was used to identify and study *cis*-acting regulatory elements in selected genes.

#### Gene expression analysis using qPCR

Real-time quantitative polymerase chain reaction (qPCR) was performed to validate selected genes in four genotypes (ICPA 2039, ICPB 2039, ICPA 2089, and ICPB 2089) differing in their ability to develop fertile pollen. qPCR analysis was carried out using Applied Biosystems 7500 Real Time PCR System with SYBR Green chemistry (Applied Biosystems, USA). The actin gene was used as an endogenous control and reactions were performed with two technical replicates and two biological replicates. The relative expression of the genes in each of the four genotypes (two male sterile and two fertile) was calculated using a modified Livak method. The Δ*C*_t_ value was calculated for each of the genes with respect to the housekeeping genes and was converted to fold change (2^−Δ*C*t^) value (Livak and Schmittgen, 2001).

#### Identification of cis-regulatory elements and splice variants

The *cis*-acting elements were identified by scanning 1500 bp upstream regions of the transcription start site of the selected genes using the PlantCARE database (Lescot *et al.*, 2002). Further, the splice junctions were identified using Finesplice (Gatto *et al.*, 2014) in all 30 samples. Those alternative splice junctions that were supported by 10 or more reads were retained and considered for further study. The identified spliced junctions were then categorized into three alternative splicing events, namely alternative 5′ donor, alternative 3′ acceptor, and exon skipping. The splicing variants were visualized using the Integrative Genomics Viewer (Robinson *et al.*, 2011) and represented in the form of a Sashimi plot (Katz *et al.*, 2015).

## Results

### Development of the *Cajanus cajan* gene expression atlas

**Fig. 1.**
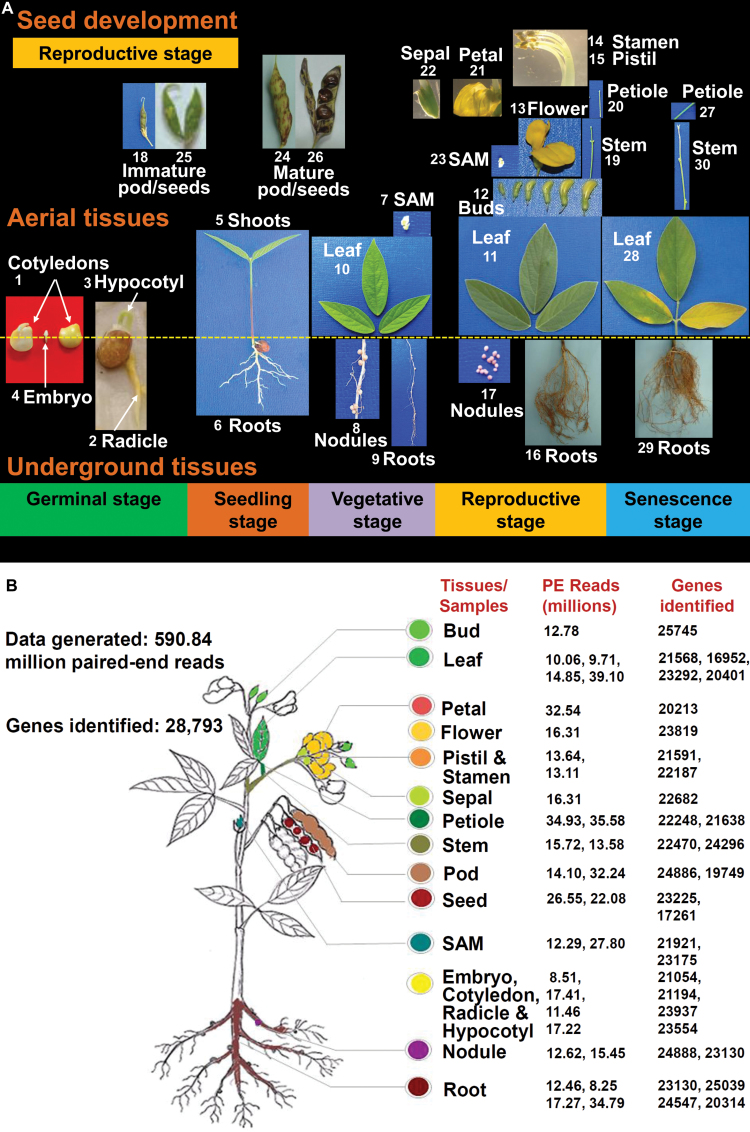
Sample details of gene expression atlas developed using Asha genotype. (A) All 30 sample used for developing the gene expression atlas of pigeonpea. The samples represent tissues/organ systems from five developmental stages of the lifecycle of pigeonpea, and also include aerial, underground and seed tissues. (B) Summary of the sequencing reads (paired-end) generated and the expressed genes identified in different tissues. Imm, immature; Mature, Rep, reproductive; Sen, senescent; Veg, vegetative.

To generate the *Cajanus cajan* gene expression atlas (CcGEA), 30 samples were collected representing all the major tissues encompassing the plant’s complete lifecycle (Fig. 1A). These 30 samples represented five major stages of plant development, namely the germinal (four tissues), seedling (two tissues), vegetative (four tissues), reproductive (16 tissues), and senescence stages (four tissues) (Table 1). Germinal tissues were represented by cotyledons, radicle, hypocotyl, and embryo. In the case of the seedling stage, shoot and root parts were separated. Vegetative tissues were composed of shoot apical meristem (SAM), nodule, root, and leaf. The majority of the samples/tissues were collected at the reproductive stages and encompassed leaf, petiole, bud, root, nodule, immature pod, immature seed, mature pod, mature seed, stem, SAM, whole flower, and distinct flower organs (i.e. sepal, petal, pistil, and stamen). Finally, at the senescence stage, petiole, leaf, root, and stem were collected. All samples were harvested in three biological replicates. For simplicity of usage, all the organs and organ system that were considered for the study were referred here as ‘tissues’ or ‘samples’.

**Table 1.** Summary of RNA-Seq data generated and genes identified in 30 plant tissues using the Illumina sequencing platform SAM, shoot apical meristem

Sample no.	Tissue	Stage	Reads (million pairs)	Mapped reads (%)	Genes identified
1	Cotyledons	Germinal	17.41	94.3	21 194
2	Radicle	Germinal	11.46	94.8	23 937
3	Hypocotyl	Germinal	17.22	95.2	23 554
4	Embryo	Germinal	8.51	95.7	21 054
5	Shoot	Seedling	10.06	95.9	21 568
6	Root	Seedling	12.46	93.0	21 357
7	SAM	Vegetative	12.29	87.5	21 921
8	Nodule	Vegetative	12.62	90.5	24 888
9	Root	Vegetative	8.25	90.6	25 039
10	Leaf	Vegetative	9.71	90.9	16 952
11	Leaf	Reproductive	14.85	94.8	23 292
12	Bud	Reproductive	12.78	94.6	25 745
13	Flower	Reproductive	16.31	95.4	23 819
14	Stamen	Reproductive	13.11	95.5	22 187
15	Pistil	Reproductive	13.64	95.6	21 591
16	Root	Reproductive	17.27	94.8	24 547
17	Nodule	Reproductive	15.45	95.2	23 130
18	Immature pod	Reproductive	14.10	95.5	24 886
19	Stem	Reproductive	15.72	95.0	22 470
20	Petiole	Reproductive	34.93	95.7	22 248
21	Petal	Reproductive	32.54	95.9	20 213
22	Sepal	Reproductive	38.42	95.1	22 682
23	SAM	Reproductive	27.80	95.5	23 175
24	Mature pod	Reproductive	32.24	93.7	19 749
25	Immature seed	Reproductive	26.55	96.1	23 225
26	Mature seed	Reproductive	22.08	95.4	17 261
27	Petiole	Senescence	35.58	95.6	21 638
28	Leaf	Senescence	39.10	95.6	20 401
29	Root	Senescence	34.79	94.2	20 314
30	Stem	Senescence	13.58	95.3	24 296

Using Illumina sequencing platform, a total of 590.84 million paired-end reads were generated from 30 samples (Fig. 1B and Table 1). The low quality reads were filtered out (see Materials and methods, RNA extraction, Illumina sequencing and data pre-processing), retaining 559.98 million paired-end reads (94.5% of the total paired-end reads) for downstream analysis. An average of 94.43% of the filtered reads mapped to the reference genome (Varshney *et al.*, 2012) using TopHat. Subsequently, a total of 28 793 genes were identified from reference guided assembly and their expression was quantified as fragments per kilobase of transcript per million fragments sequenced (FPKM) using Cufflinks (Supplementary Table S1). Gene expression profiling of 28 793 genes within different tissues and stages identified 27 132 genes with higher abundance (FPKM>1) and 1661 genes with lower abundance (FPKM<1). These results demonstrated sufficient sequencing coverage across the pigeonpea genome. In order to generate a comprehensive dataset for further analyses, genes were annotated based on previously annotated pigeonpea gene models (Varshney *et al.*, 2012). Gene information such as the protein domain (UNIPROT), the putative cellular localization, the orthologous gene annotations in other species, the gene ontology (GO) IDs and names, and the putative corresponding pathways of each of these genes are indicated in Supplementary Table S1. Expression values of all annotated genes from the 30 samples were finally log_2_-transformed (Supplementary Table S1).

### Analysis of gene expression clusters

**Fig. 2.**
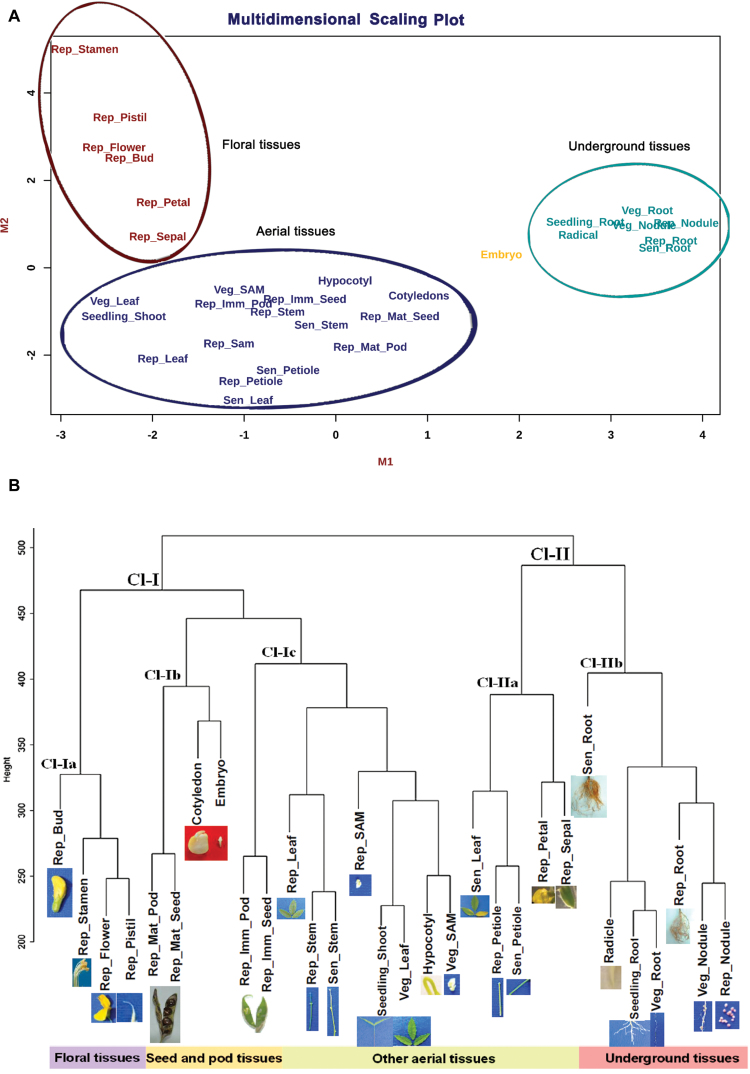
Sample clustering of 30 samples based on expression values. (A) Multi-dimensional scaling (MDS) plot of all 30 tissues with scaling performed in two dimensions, M1 and M2. (B) Sample clustering diagram to detect outliers with cutoff height shown on the *y*-axis. The height scale is the distance metrics between the clusters, and samples displaying low merging height are highly related. Sample clustering showed two clades, Cl-I representing aerial and Cl-II representing aerial and underground tissues. Aerial clade Cl-I is composed of three subclades, namely floral, seed and different aerial tissues. Cl-II is composed to two distinct clades, aerial and underground. The sample clustering did not reveal any outliers. Veg, vegetative; Rep, reproductive; SAM, shoot apical meristem; Sen, senescent.

To evaluate the quality of the generated gene expression atlas, a multi-dimensional scaling (MDS) analysis was first performed using the global expression dataset across the 30 samples. A clear repartition of the dataset in four major groups was observed, which represented the origin of the tissues, with aerial, underground, floral and embryo groups (Fig. 2A). Simultaneously, Weighted Gene Co-expressed Network Analysis (WGCNA; Langfelder and Horvath, 2007, 2008), an R package, was utilized to identify sample outliers. Thirty samples clustered into two major clades, designated as clade I (Cl-I) and clade II (Cl-II) (Fig. 2B). Clade Cl-I bifurcated into three subclades, Cl-Ia corresponding to floral tissues, Cl-Ib corresponding to mature seed tissues, and Cl-Ic corresponding to aerial tissues including shoot primordial tissues such as hypocotyl, SAM and seedling. On the other hand, clade Cl-II comprised two subclades, Cl-IIa, which consisted of aerial tissue representing the reproductive and senescent stages, and Cl-IIb, which included all underground tissues (radicle, root, and nodule). As expected, there was no outlier detected from our set of samples, which validated the tissue sample preparation.

From the transcriptomic dataset, a total of 1044 stably expressed genes have been identified within all tissues (i.e. displaying a coefficient of variation below 30%; Supplementary Table S2). The catalogue of stably expressed genes represents a resource for identification of reference or housekeeping genes, which are necessary for comparative gene expression analysis to normalize transcript expressions due to developmental or environmental fluctuations. In the dataset, 62 stably expressed genes within all the tissues with a coefficient of variation (CV) below 20% were identified (Supplementary Table S2). These genes were mainly annotated as involved in basic cell functions such as RNA machinery (*C.cajan_18478*, *C.cajan_31580*, *C.cajan_19893*, *C.cajan_16707*), cell cycle machinery factors (*C.cajan_10165*, *C.cajan_16931*, *C.cajan_25222*) and actin-related protein (*C.cajan_31922*). Based on the large diversity of analysed tissues in this study, it was difficult to identify genes displaying a CV lower than 10%. However, based on our comparative gene expression experiment, this catalogue of stably expressed genes could be refined to study specific tissues and/or stages. For instance, in the case of an experiment based on specific plant organs, 50 potential reference genes displaying a CV below 10% were identified for the study focused on underground tissues, 43 genes for the study of pod and seed tissues, and three genes for the study of aerial tissues (Supplementary Table S2).

### Gene expression patterns across different tissues

**Fig. 3.**
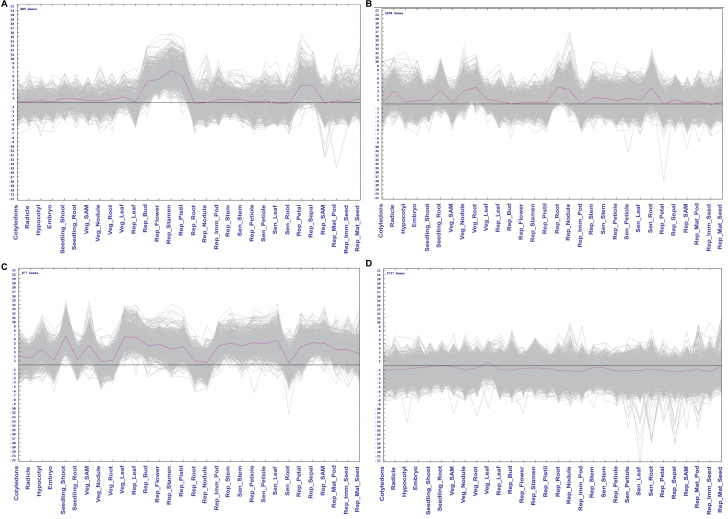
*k*-Means cluster analysis. (A) Cl-II depicting floral tissue-specific gene expression. (B) Cl-IV depicting underground tissue-specific gene expression. (C) Cl-V depicting aerial tissue-specific gene expression. (D) Cl-X depicting senescent tissue-specific gene expression.

The 28 793 genes were grouped into ten clusters (Cl-I to Cl-X) based on the *k*-means clustering algorithm with Euclidean distance as the similarity criterion using Multiexperiment Viewer (MeV; Howe *et al.*, 2010; Supplementary Fig. S2). The optimum cluster number has been determined by plotting the sum of squared error values against the different values of *k* (Everitt and Hothorn, 2005). Cl-I, -III, -VI, -VII, -VIII, and -IX exhibited genes with constitutive expression across the tissues, among which Cl-I, -III, -VII, and -IX showed high gene expression. Interestingly, Cl-II comprised 685 genes and displayed floral-tissue preferential expression (Fig. 3A) including 14 transcription factors (TFs), 11 cytochrome P450, and ten L-ascorbate oxidase homologs among others. This cluster consisted of several transcription factors previously reported as developmental regulators, such as floral homeotic proteins *AGAMOUS*, *APETALA 1*, *GLOBOSA* and *DEFICIENS* (Egea‐Cortines *et al.*, 1999; Lohmann and Weigel, 2002). Other putative regulators were AGAMOUS-like MADS-box proteins AGL18 and AGL19, zinc finger proteins CONSTANS-like 3 and 4, NAC25, putative WRKY42, MYB113, SEPALLATA 2–3, and HAT5 (see corresponding gene annotations in Supplementary Table S3).

Similarly, Cl-IV (1459 genes; Fig. 3B) and Cl-V (877 genes; Fig. 3C) exhibited underground and aerial preferential expression, respectively. The underground-preferential cluster (Cl-IV) included those encoding 1-aminocyclopropane-1-carboxylate oxidase, ABA 8′-hydroxylase 1, cytokinin synthase 5, cytokinin dehydrogenase 3, agamous-like MADS-box protein AGL12, AP2-like ethylene-responsive transcription factor AIL6, cationic peroxidase 1, cellulose synthase A, cysteine-rich receptor-like protein kinase 6, calcium/calmodulin-dependent serine/threonine-protein kinase DMI-3, dehydration-responsive element-binding protein 1B, dirigent protein, and disease resistance response protein among others (Supplementary Table S4). This gene set revealed that phytohormone regulation, cell-wall modification, defense response and signaling are crucial mechanisms in underground tissues (i.e. Seedling_Root, Veg_Root, Veg_Nodule, Rep_Root, Rep_Nodule and Sen_Root). On the other hand, the aerial-preferential cluster (Cl-V) was composed of genes encoding ABI5-like protein 4, phytochrome-interacting TFs PIF4, auxin-induced protein, chlorophyll a/b binding protein 13, cytochrome P450, GATA transcription factor 9, GDSL esterase/lipase APG, homeobox-leucine zipper protein ANTHOCYANINLESS 2, putative axial regulator YABBY 2, etc. Transcription factor PIF4 and ABI5 are involved in inducing leaf senescence (Sakuraba *et al.*, 2014), but might have a similar function in petioles, stem and other aerial tissues as well. Other important TFs identified in this aerial preferential cluster included several bHLHs (e.g. *C.cajan_28596*, *C.cajan_09519*, *C.cajan_12003*), EN133, EN67, DIVARICATA, HY5 homolog, SCREAM, MYB86, and RADIALIS (Supplementary Table S5). Cl-X (1830 genes; Fig. 3D) showed down-regulated gene expressions preferentially in senescing tissues (i.e. Sen_Leaf, Sen_Root, Sen_Stem and Sen_Petiole). Genes belonging to this cluster encoded cyclin-dependent kinase B2-2, DNA (cytosine-5)-methyltransferase CMT3, DNA replication complex GINS protein SLD5, E2F transcription factor-like E2FE, mitotic checkpoint protein BUB3.3, histone-lysine *N*-methyltransferase ASHR3, and meiotic nuclear division protein 1 homolog. Down-regulation of these genes could reflect impairment of DNA replication and the cell cycle. Other processes indicating cell damage included signaling and defense genes such as F-box protein, galactose oxidase, GDSL esterase/lipase, germin-like protein subfamily 3, heparanase-like protein 3, homeobox-leucine zipper protein HDG11, LRR receptor-like serine/threonine-protein kinase GSO1, peroxidase 52, potential protein lysine methyltransferase SET5, protein IQ-DOMAIN 14, sugar transport protein 14, Myb-related protein 86, and probable inactive leucine-rich repeat receptor-like protein kinase (see corresponding gene annotations in Supplementary Table S6).

### Differentially expressed genes and identification of senescence-related genes

**Fig. 4.**
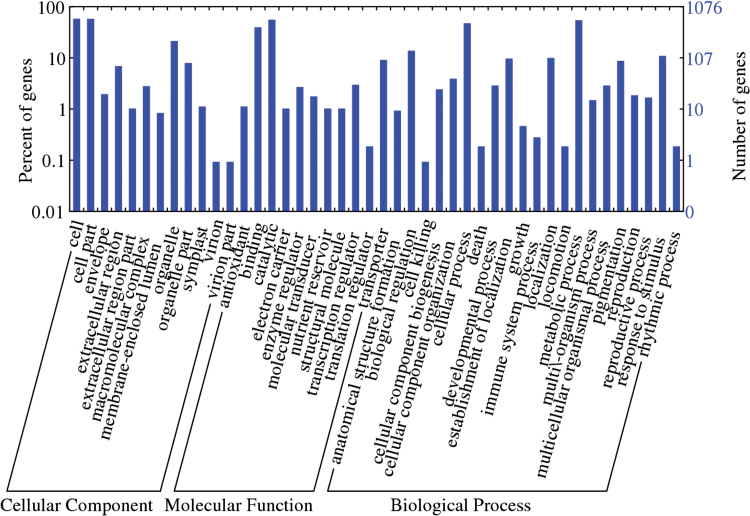
Summary of level 2 GO terms assigned by Blast2GO. Gene Ontology classification of the identified DEGs into three major categories, cellular component, molecular function and biological process. The result is summarized in a histogram with the left *y*-axis representing the percentage of a genes and the right *y*-axis indicating the number of genes falling into level 2 classification (*x*-axis).

To further validate and analyse the CcGEA, pairwise comparisons were performed among 30 samples using all different combinations to identify differentially expressed genes (DEGs). A unique set of 1076 DEGs showed significant differential expression, either induced or repressed depending on the tissue (Supplementary Table S7). For conciseness, tissue samples were represented as Stage_tissue name, ‘Veg’ for vegetative, ‘Rep’ for reproductive, ‘Sen’ for senescence, ‘Mat’ for mature and ‘Imm’ for immature tissues. Comparisons were named as the sample name followed by *versus* then the samples being compared. For example, the comparison Veg_SAM *vs* Veg_Leaf represents the DEGs identified by comparing vegetative leaf to vegetative SAM. The majority of the DEGs were identified between Rep_Petiole *vs* Sen_Petiole (89 genes), followed by Rep_Bud *vs* Immature_Pod (79 genes) and Sen_Stem *vs* Sen_Petiole (53 genes) and Veg_Root *vs* Seedling_Root (49 genes). Differentially expressed genes showed up to 10- to 14-fold expression differences in different pairwise comparisons, which include beta-conglycinin (*C.cajan_28781*) and late embryogenesis abundant protein (LEA, *C.cajan_03928*) in mature seeds, sugar-binding proteins (*C.cajan_34645*) in mature pods, and glycerol-3-phosphate acyltransferase 6 (*C.cajan_45202*), acid beta-fructofuranosidase (*C.cajan_01573*), and cinnamoyl-CoA reductase 1 (*C.cajan_29356*) in petals. Further, GO annotations of 1076 DEGs identified more than 50% of genes mainly involved in metabolic process (biological processes), binding, catalytic and cellular process (molecular functions), and constituting cell and cell parts (cellular components). Few genes were also found to be involved in other biological processes such as cellular processes, response to stimuli, and pigmentation (Fig. 4).

The dynamics of gene expression during the course of development in pigeonpea were studied to show the potential of the CcGEA to answer biological questions related to temporally or spatially regulated genes. We focused on DEGs between leaf at the vegetative and senescence stages (Veg_Leaf *vs* Sen_Leaf), and similarly for root tissues (Veg_Root *vs* Sen_Root), in order to identify senescence-related genes. In the senescing leaf (Sen_Leaf), 29 genes were identified encoding plant U-box protein 18 (*C.cajan_02471*), small chloroplastic heat shock protein (*C.cajan_03228*), desiccation-responsive protein 29B (*C.cajan_07296*), universal stress protein Slr1101 (*C.cajan_07683*), calcium permeable stress-gated cation channel 1 (*C.cajan_09319*), galactinol synthase 2 (*C.cajan_10278*), transcription factor PIF3 (*C.cajan_10677*), MYB48 (*C.cajan_13565*), homeobox-leucine zipper protein ATHB-12 (*C.cajan_10940*), MUD21-2 protein (*C.cajan_12676*), endoglucanase (*C.cajan_13234*), and senescence-related gene 1 (*C.cajan_14735*). These genes showed negligible expression in the Veg_Leaf. Similarly, 39 genes were identified exclusively in Sen_Root, including cation transport regulator-like protein 2 (*C.cajan_09112*), DnaJ homolog subfamily B (*C.cajan_10265*), PRA1 family protein B4 (*C.cajan_17674*), glutathione *S*-transferase (*C.cajan_19173*), signal recognition particle receptor subunit α homolog (*C.cajan_22178*), ERF096 (*C.cajan_22554*), remorin (*C.cajan_26491*), probable aquaporin NIP7-1 (*C.cajan_28406*), vignain (*C.cajan_28469*), squamosa promoter-binding protein 1 (*C.cajan_31328*), β-galactosidase 13 (*C.cajan_32927*), agamous-like MADS-box protein AGL11 (*C.cajan_36667*), probable LRR receptor-like serine/threonine-protein kinase (*C.cajan_38944*), signal peptidase complex subunit 3B (*C.cajan_39695*), and heat stress transcription factor B-2b (*C.cajan_44684*).

**Fig. 5.**
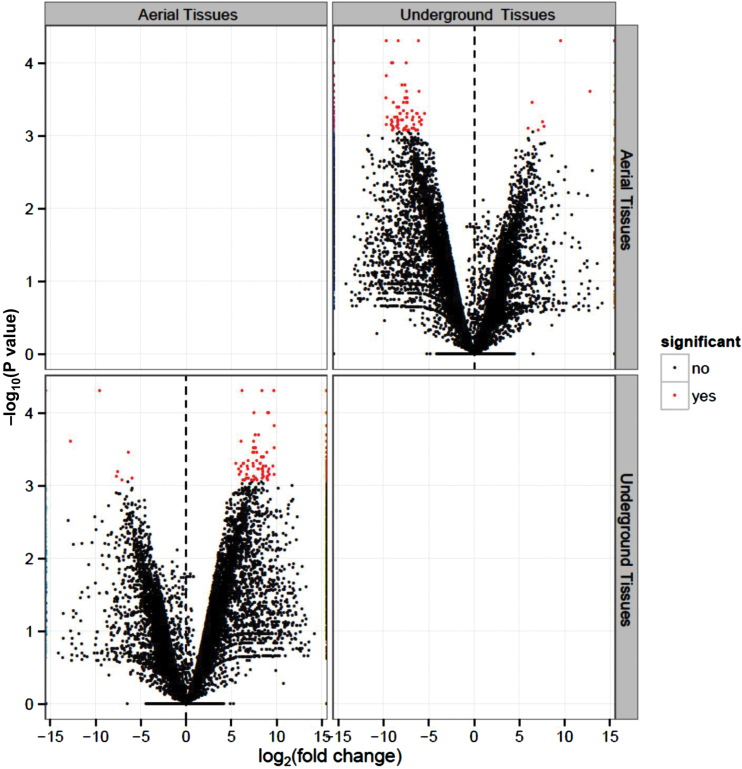
Volcano plot showing significant genes that were differentially expressed between aerial and underground tissues. Analysis and visualization of significant differentially expressed genes was performed using CummeRbund. Each spot represents a gene that has been plotted between log2 (fold change) on the *x*-axis and –log_10_ of the *P*-value on the *y*-axis. Red dots represent significantly regulated genes (either up- or down-regulated) and black dots represent non-significant genes.

Similarly, spatially regulated genes between aerial and underground tissues were also studied. In order to examine the gene expression patterns in these two types of tissues, all the aerial tissues and all the underground tissues were pooled. As expected, a volcano plot depicted significant up- and down-regulation of DEGs between aerial and underground tissue samples (Fig. 5). All up-regulated genes were plotted on the right hand side of the dotted line passing through the center of the volcano, while down-regulated genes were plotted towards the left hand side. A total of 75 DEGs including 68 significantly up-regulated and seven down-regulated with log_2_ fold change values ≥5 or ≤–5, respectively, were identified (Fig. 5). Among aerial tissues, highly expressed genes included those encoding mainly photosynthetic apparatus-related chloroplastic proteins (*C.cajan_01316*, *C.cajan_13889*, *C.cajan_13832*, etc.) and cell-wall modifying enzymes (*C.cajan_42632*), along with other proteins such as late embryogenesis abundant protein D-29 (*C.cajan_03928*), basic 7S globulin (*C.cajan_10207*), and oxygen-evolving enhancer protein 3-2 (*C.cajan_37794*). Other enzymes involved in photorespiratory carbon metabolism and wax biosynthesis have also been identified in aerial tissues, such as serine-glyoxylate aminotransferase (*C.cajan_22602*) (Somerville and Ogren, 1980) and 3-ketoacyl-CoA synthase 17 (*C.cajan_28231*) (Todd *et al.*, 1999), respectively. Among the underground tissue samples, DEGs encoding leghemoglobin (*C.cajan_22417*), sugar transport protein 13 (*C.cajan_07532*), subtilisin-like protease SDD (*C.cajan_02637*), probable 2-oxoglutarate-dependent dioxygenase AOP1.2 (*C.cajan_00158*), and cytochrome P450 78A5 (*C.cajan_19316*) were highly expressed in nodules, while cytokinin dehydrogenase 3 was over-expressed in the radicles. Subtilisin-like proteases and leghemoglobins have been suggested to have widespread function during early stages of nodule symbioses (Ribeiro *et al.*, 1995; Szczyglowski *et al.* 1997).

### Tissue-specifically expressed genes

**Fig. 6.**
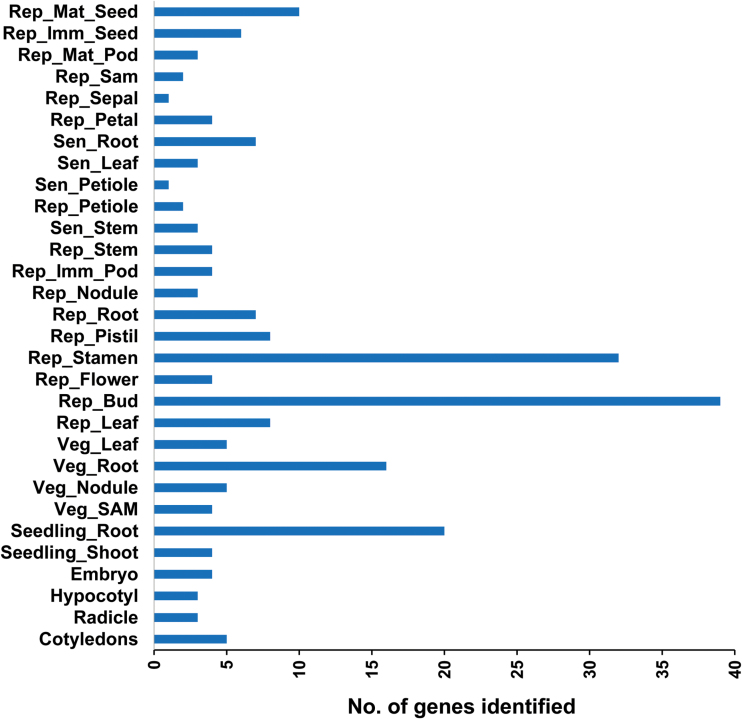
Genes identified as being specifically expressed in each of the 30 tissue samples.

Apart from DEGs, 220 genes were identified with specific expression in exclusively one tissue (Fig. 4 and Supplementary Table S8). Tissues displaying the highest number of specifically expressed genes were bud (39), stamen (32) and seedling root (20) (Fig. 6). Various TFs have been specifically expressed in different tissues, including MYB-like protein J (*C.cajan_48299*) from bud, RADIALIS (*C.cajan_31277*) from leaf (reproductive stage), and GATA transcription factor 27 (*C.cajan_17218*) from immature seeds (5 days after anthesis). Several other genes were also identified, such as those encoding BOBBLER 2, PHD finger protein ALFIN-LIKE 1, and plantacyanin in bud, superoxide dismutase and auxin-induced protein 6B in nodules (vegetative stage), FAR-RED IMPAIRED RESPONSE 1 protein in stamen, polygalacturonase in pistil, and sucrose synthase 2 in mature seeds (30 days after anthesis), apart from several retrovirus-related proteins. Defense-related proteins such as defensin-like protein 19 and defensin-like protein 244 were specifically expressed in root (reproductive stage) and immature pods (5 days after anthesis), respectively. A complete list of tissue-specific genes is provided in Supplementary Table S8.

### Identification of flower-related genes using a network analysis approach

**Fig. 7.**
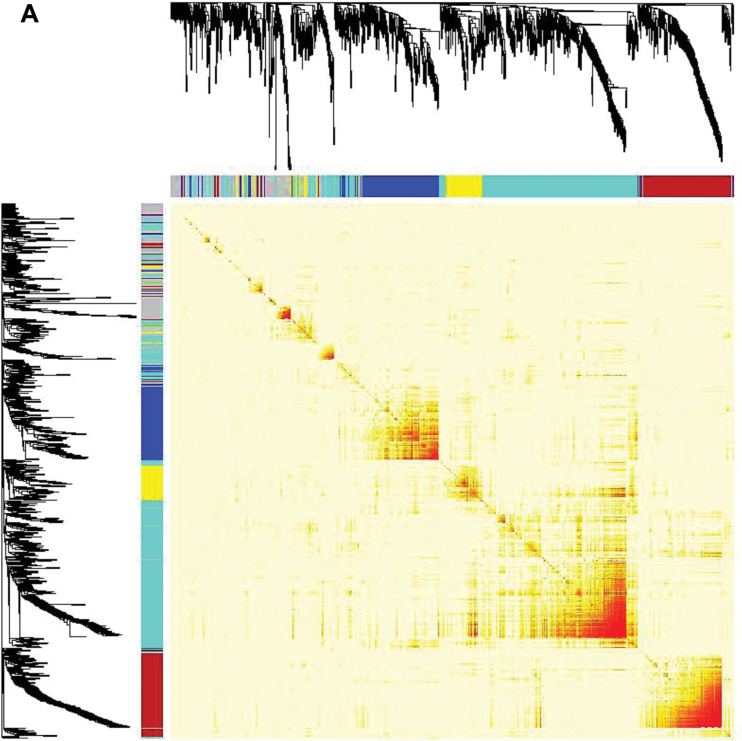

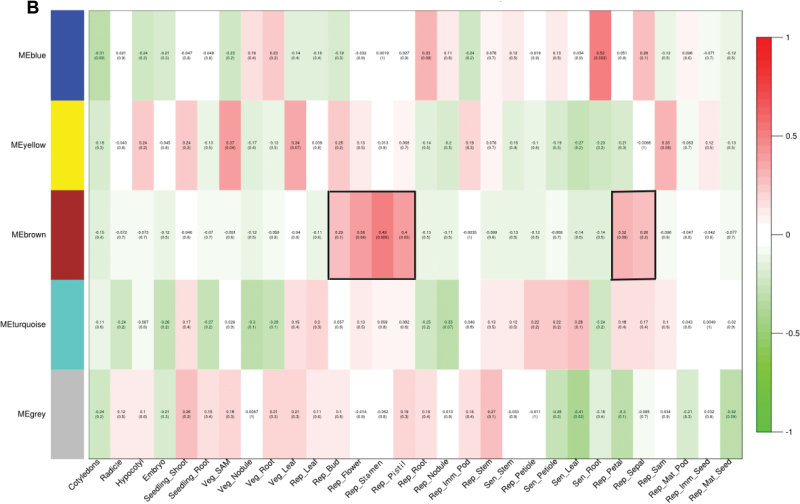

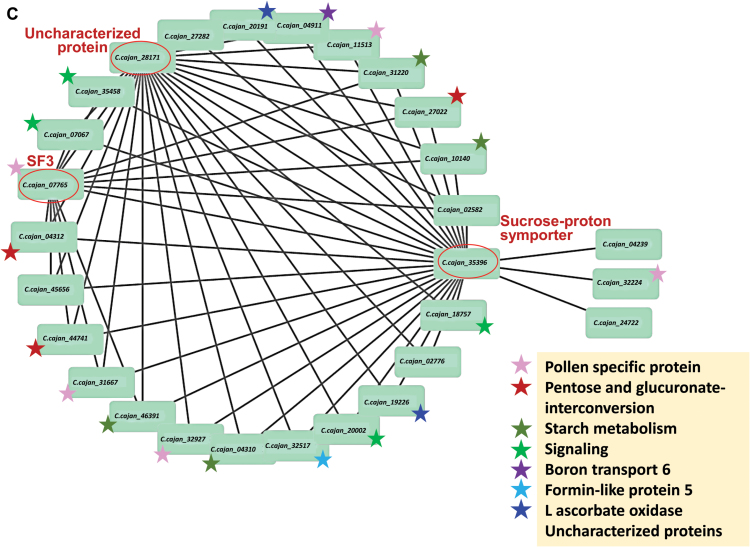

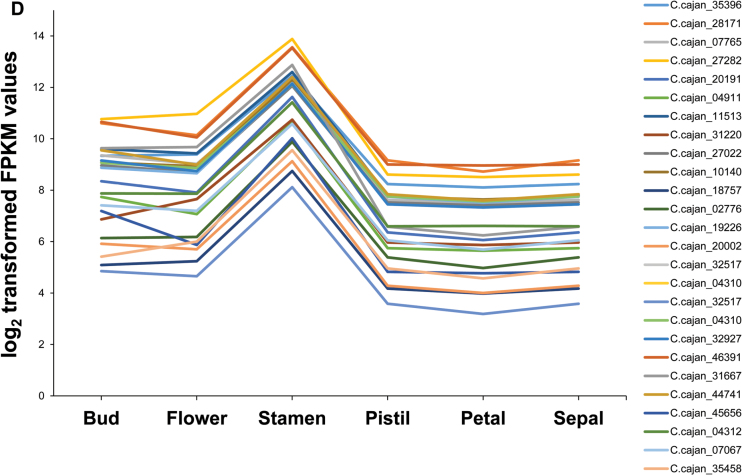
Correlation matrix of the differentially expressed genes. (A) Hierarchical clustering of the topological overlap matrix (TOM) of the differentially expressed genes. The color bar at the top and the left of the heatmap shows the module assignment obtained from WGCNA. The intensity of red denotes the absolute value of Pearson’s correlation coefficients between the expression profiles of all pairs of differentially expressed genes, which were transformed into network connection strengths. Rows and columns represent genes and are symmetrical. (B) Module to tissue association. Each row represents a module and each column represents a sample. Red represents a positive correlation, whereas green represents a negative correlation between the module and the sample. (C) Correlation network of the floral transcriptome. (D) Graph depicting co-expressed genes in all the six floral tissues (bud, flower, stamen, pistil, petal, and sepal).

A group of genes identified by expression patterns that are tightly interconnected constitutes a module that is most likely to be involved in common biological functions. In order to understand the complex biological networks involved in the different developmental processes, a systems biology approach using WGCNA was used to analyse DEGs from different tissue combinations. A correlation matrix was generated among the 30 tissues using a set of 1076 DEGs resulting in three major modules, turquoise (containing 400 genes), blue (208 genes) and brown (197 genes). Here, modules are referred to the distinct groups formed by the clustering of genes, and each module has been designated by an arbitrary color to distinguish between them (Fig. 7A). Module to tissue association was measured and resulted as a close relationship between the turquoise module and aerial tissues, whereas blue and brown modules were associated with underground and floral tissues (i.e. bud, flower, pistil, and stamen), respectively. As observed by Pearson’s correlation coefficients and *P*-values, the strongest expression association was measured in the brown module, especially in the floral tissues (marked with a red square in Fig. 7B). Therefore, a weighted correlation network for genes belonging to the brown module was visualized using Cytoscape. 

The circular network depicted 28 genes represented by nodes, interconnected with edges (connecting lines between genes) to identify three highly connected genes, referred to as ‘hub’ genes. WGCNA defines co-expression networks as weighted gene network, where the nodes correspond to gene expression profiles, and edges are determined by pair-wise correlations between gene expressions. Genes within the co-expression module that display high connectivity form the ‘highly connected genes’ referred to as ‘hub genes’ (Langfelder and Horvath, 2008). The hub genes identified in the co-expression network were annotated as encoding a sucrose-proton symporter 2 (*C.cajan_35396*), a pollen specific SF3 protein (*C.cajan_07765*) and an uncharacterized protein (*C.cajan_28171*). *C.cajan_35396* gene encoding a putative H^+^ symporting sucrose transporter protein 2 has been suggested to be involved during pollen maturation in mediating sucrose uptake in pollen grains (Lemoine *et al.*, 1999). In the co-expression network generated, each of the two hub genes (*C.cajan_35396* and *C.cajan_28171*) was connected to all the other 27 genes, which encoded serine threonine protein kinases (*C.cajan_18757*, *C.cajan_20002*, *C.cajan_07067*), pectinesterase inhibitors (*C.cajan_10140*, *C.cajan_04310*, *C.cajan_46391*), pectate lyase 3 (*C.cajan_ 44741*, *C.cajan_27022*), pollen-specific proteins (*C.cajan_02582*, *C.cajan_07765*, *C.cajan_11513*), Olee1-like (*C.cajan_31667*, *C.cajan_32224*), L-ascorbate oxidase homolog (*C.cajan_19226*, *C.cajan_20191*), ATPase 8 (*C.cajan_45656*), β-galactosidase 13 (*C.cajan_32927*), polygalacturonase (*C.cajan_04312*), phosphatidylinositol transfer protein (*C.cajan_35458*), boron transporter 6 (*C.cajan_04911*), formin-like protein 5 (*C.cajan_32517*), aldose 1-epimerase (*C.cajan_31220*), and uncharacterized proteins (C*.cajan_ 04239*, *C.cajan_24722*, *C.cajan_02776*, *C.cajan_27282*). Among these genes, L-ascorbate oxidase, β-galactosidase, polygalacturonase homolog and sucrose transporter were reported to be pollen-specific genes (Masuko *et al.*, 2006). *C.cajan_07765*, annotated as SF3 protein, displayed a high and specific expression in floral tissues including bud, flower, pistil, stamen, petal, and sepal. This gene has been previously reported as a TF regulating the expression of late pollen genes (Baltz *et al.*, 1992*a*) and has been found to be connected to 13 other genes in the co-expression network (Fig. 7C). All the genes were co-expressed exclusively in floral tissues (bud, stamen, pistil, petal, and sepal) with high gene expression in stamen (Fig. 7D), which validated this approach to identifying a gene network of flower development-related genes.

#### Expression analysis of flower-related genes

**Fig. 8.**
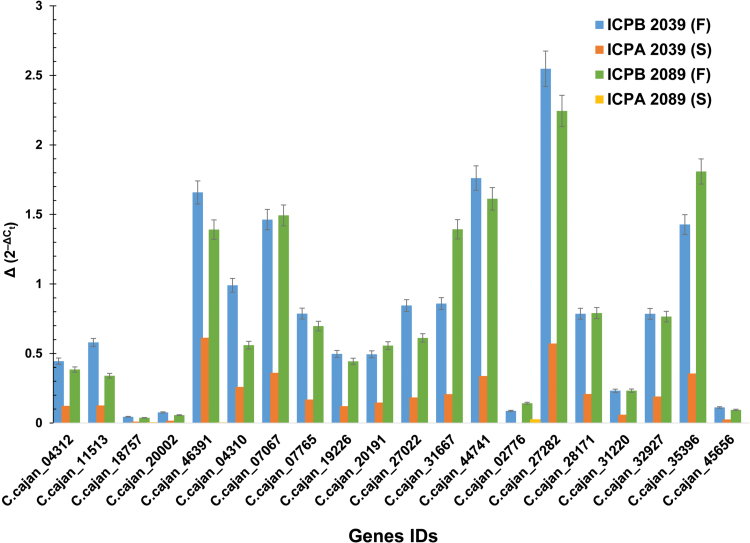
Expression analysis of flower-related genes using qPCR. Expression of the 25 genes of the floral gene network was validated using qPCR in the flowers of two sets of male sterile genotypes (ICPA 2039 and ICPA 2089) and their fertile counterparts (ICPB 2039 and ICPB 2089). All these genes showed a much lower expression in the male sterile genotypes than in the fertile genotypes. S, sterile; F, fertile.

Expression of 25 genes (Supplementary Table S9) belonging to the floral gene network (circular-closed network, Fig. 7C) was analysed in the floral tissues using qPCR. Gene expression was studied in four pigeonpea genotypes, two of which were male sterile (ICPA 2039 and ICPA 2089) and two fertile (ICPB 2039 and ICPB 2089). These genotypes were used for an A_4_-based hybrid breeding system in pigeonpea (Saxena *et al.*, 2011). Out of 25, 20 genes showed marked differences in their expression among the male sterile and fertile counterparts. These genes showed much lower expression (0 to 0.6-fold change) in the male sterile genotypes compared with the fertile genotypes (ICPB 2039) (Fig. 8). A more than 1.4-fold increased expression of *C.cajan_46391*, *C.cajan_44741*, *C.cajan_27282* and *C.cajan_35390* was observed in both fertile genotypes, namely ICPB 2039 and ICPB 2089, when compared with their male sterile counterparts. *C.cajan_27282*, an uncharacterized protein, showed 2.5-fold induced expression in the fertile genotypes (ICPB 2039) while in the sterile genotypes the expression was found negligible (0.5-fold).

#### Promoter analysis of flower-related genes

Sequence analyses of the promoter regions of these co-expressed genes identified a majority of *cis*-acting elements to be involved in light-responsiveness, in addition to the elements required for seed-specific regulation, endosperm expression and phytohormone responsiveness. In 25 flower-related genes, the promoter sequences could be identified and contained 243 light-responsive *cis*-elements, suggesting a strong stimulus-dependent expression, particularly in response to light. In addition, phytohormone regulation of these genes was suggested due to the presence of multiple methyl jasmonic acid (MeJA), salicylic acid (SA), gibberellin (GA) and abscisic acid (ABA) responsive elements. Genes that were validated using qPCR also showed the presence of light responsive, circadian control, MeJA, SA, auxin, ABA, and endosperm-responsive sequence elements (Supplementary Table S10).

#### Splice variant study of flower-related genes

**Fig. 9.**
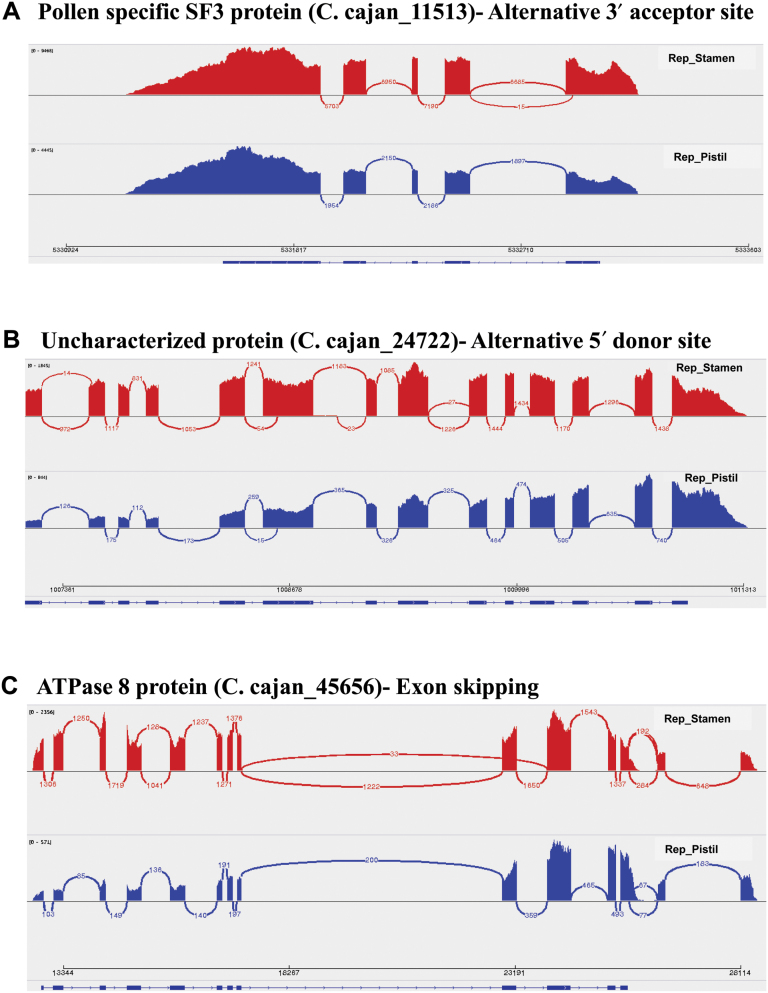
Alternative splicing in three flower-related genes. Three examples of alternative splicing variants have been shown in Rep_Stamen samples compared with Rep_Pistil using a Sashimi plot. The three flower-related genes, namely *C.cajan_11513*, *C.cajan_24722*, and *C.cajan_45656*, have shown an alternative splicing event in Rep_Stamen (displayed in blue) with respect to Rep_Pistil (displayed in red). Alternative 3′ acceptor site, alternative 5′ donor site and exon skipping have been found in *C.cajan_11513*, *C.cajan_24722*, and *C.cajan_45656*, respectively. The numbers in the figure denote the read coverage supporting the alternative splicing.

Alternative splicing (AS) events were studied in all the 28 genes belonging to a floral gene network across all the 30 samples. Overall, 18 AS events were identified among eight genes, namely *C.cajan_11513*, *C.cajan_31667*, *C.cajan_35458*, *C.cajan_45656*, *C.cajan_35396*, *C.cajan_18757*, *C.cajan_27022* and *C.cajan_24722*. These AS events consisted of an alternative 3′ acceptor site, an alternative 5′ donor site, exon skipping, and alternative 3′ and 5′ splice sites. All the eight genes showed splicing events in floral tissues (bud, flower, stamen, pistil, sepal, and petal), while *C.cajan_18757* also showed an alternative 5′ donor site in Immature seed, Immature pod, Sen_Petiole, and Rep_SAM (Supplementary Table S11). The ‘hub’ gene encoding sucrose-proton symporter 2 (*C.cajan_35396*) and two other genes encoding a pollen-specific SF3 protein (*C.cajan_11513*, Fig. 9A) and an uncharacterized protein (*C.cajan_24722*, Fig. 9B) have AS of ‘alternative 3′ acceptor site’ preferentially in stamen. On the other hand, two splice variants (alternative 5′ donor site and exon skipping) have been identified for *C.cajan_45656* preferentially found in bud and stamen (Fig. 9C), whereas an alternative 3′ acceptor site and alternative 5′ donor site has been observed exclusively in petals and sepals for the gene *C.cajan_31667* encoding an Olee 1-like protein. In all the six floral tissues, an alternative 3′ and 5′ splice site was also identified for *C.cajan_35458* and *C.cajan_32927*. Three examples of AS events preferentially found in Rep_Stamen for three different genes are shown in comparison with Rep_Pistil (Fig. 9A–C). The AS events, namely alternative 3′ acceptor site (Fig. 9A), alternative 5′ donor site (Fig. 9B) and exon skipping (Fig. 9C), have been identified for the genes *C.cajan_11513*, *C.cajan_24722*, and *C.cajan_45656*, respectively.

### Effect of epitranscriptome in tissues

**Fig. 10.**
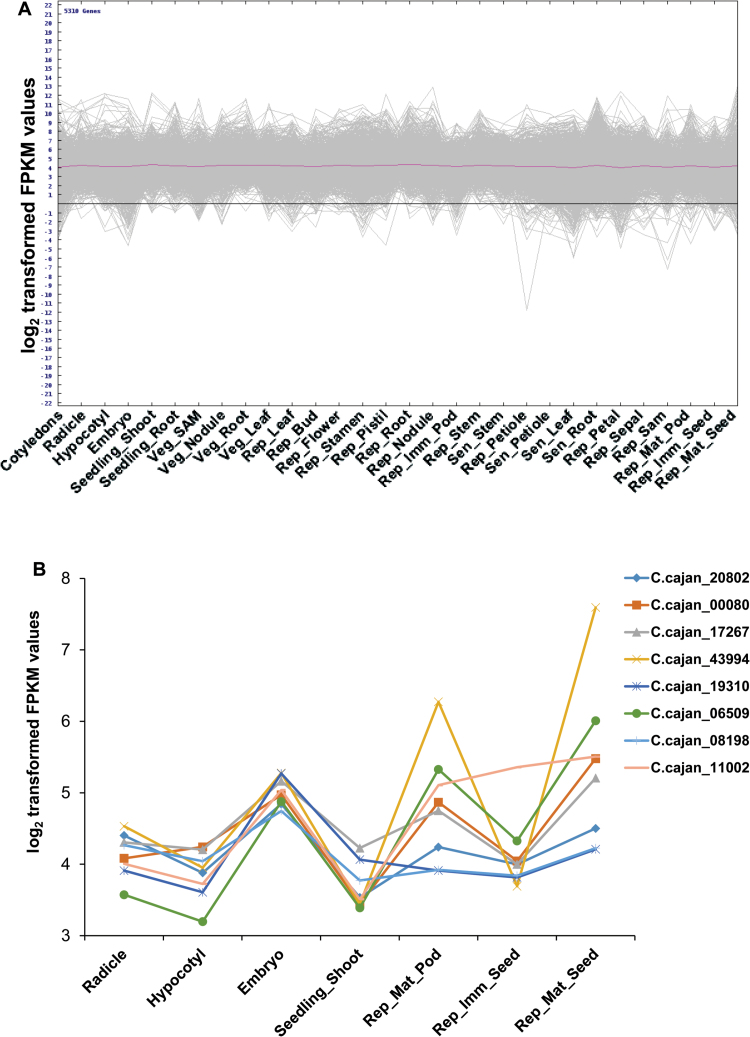
Co-expressed genes displaying epitranscriptomic regulation. (A) Co-expressed genes involved in epitranscriptomics. (B) Expression of epitranscriptomics related genes in radicle, hypocotyl, embryo, seedlings, and immature and mature seeds and pods.

In order to understand post-transcriptional regulation, an attempt has been made to identify the orthologs of m6A methyltransferase (MTA), FKBP12-interacting protein of 37 kDa (FIP37) and YT521-B homology (YTH)-domain containing protein in pigeonpea. These genes were previously reported to be involved in mRNA methylation, recognition and demethylation in Arabidopsis (Zhong *et al.*, 2008). As a result, eight orthologs were identified in pigeonpea, namely MTA70-like (*C.cajan_20802*), FIP-37 (*C.cajan_00080*), YTH domain-containing family protein 1 (*C.cajan_17267*), YTH domain-containing family protein 2 (*C.cajan_43994*), α-ketoglutarate-dependent dioxygenase alkB (*C.cajan_19310*), α-ketoglutarate-dependent dioxygenase alkB homolog 6 (*C.cajan_06509*), alkylated DNA repair protein alkB homolog 8 (*C.cajan_08198*) and α-ketoglutarate-dependent dioxygenase AlkB homolog (*C.cajan_11002*). All these genes displayed a similar pattern of expression within the 30 tissues and belonged to cluster VI (Fig. 10A). However, all eight genes displayed a higher abundance in developing tissues such as Radicle, Hypocotyl, Embryo, Seedling_Shoot, Rep_Mat_Pod, Rep_Imm_Seed and Rep_Mat_Seed, (i.e. log_2_ transformed FPKM ≥3), which suggested the involvement of post-transcriptional regulation in these developing tissues (Fig. 10B). Interestingly, an increase in the expression of these genes in Embryo, Rep_Mat_Pod and Rep_Mat_Seed has been observed, implying a possible intense post-transcriptional regulation in these specific tissues.

## Discussion

In this study, a comprehensive gene expression atlas of pigeonpea (CcGEA) has been developed, which catalogued more than 28 000 genes that were expressed in 30 diverse tissues of the plant and at five different developmental stages. This comprehensive dataset will enhance the present understanding of the genes involved in various regulatory and metabolic processes, which could directly impact important agronomic traits. With the recent advances in genomics research, GAB has accelerated precision and efficiency of breeding in many crops (Varshney *et al.*, 2013; Kole *et al.*, 2015). Development of the CcGEA together with the available genome sequence and other genomic resources offers an opportunity for pigeonpea researchers to not only solve biological questions, but also facilitate the GAB for pigeonpea improvement.

The RNA-seq data were analysed by pairwise comparison of all the samples between one another to identify the differentially expressed genes and also by clustering genes based on different algorithms. The best way to look into the complex biological and metabolic processes is to cluster genes based on their expression pattern and analyse them. By clustering genes, it would be possible to understand the co-regulated and functionally related genes. *k*-means algorithm based clustering identified clusters related to aerial, underground and senescing tissues, in addition to the DEGs identified by pairwise comparison between tissues and stages. A more complex co-expression-based gene cluster analysis (i.e. WGCNA) was further used to identify functional modules based on the assumption that each module contains genes involved in similar biological function (Holter *et al.*, 2001; Hasty *et al.*, 2001; Shen-Orr *et al.*, 2002; Mutwil *et al.*, 2010). A similar approach was effectively utilized to uncover complex and novel networks involved in strawberry flower development (Hollender *et al.*, 2014). Instead of looking into the individual gene, studying the module or cluster of genes that are expressed in a similar fashion would be more informative.

To demonstrate the usefulness of the CcGEA for identifying candidate genes for key agronomic traits, as an example, a module related to flower development (the brown module) identified by gene network analysis has been studied. The brown module revealed pigeonpea genes involved in late pollen maturation, pollen tube formation and fertilization. The ‘hub’ gene of the network (*C.cajan_07765*) was a pollen-specific *SF3* gene, which is a developmentally regulated TF, well documented in Arabidopsis to play a role in expression of late pollen genes, pollen tube formation and fertilization events (Baltz *et al.*, 1992a, b). Another ‘hub’ gene, *C.cajan_35396* codes for a H^+^-symporting sucrose transporter protein 2, typically named SUC2 protein, responsible for loading sucrose into sink cells such as developing pollen (Sauer, 2007) and other floral tissues. These genes were predicted to regulate directly or indirectly others genes in the network. Others genes such as *C.cajan_44741*, *C.cajan_04312*, and *C.cajan_27022* encoded proteins involved in pentose and glucuronate interconversion, also known to have a significant role in mature pollen development (Ma *et al.*, 2012). A putative boron transporter (*C.cajan_04911*), important for maintaining boron homeostasis, is critical for pollen viability and ability to accumulate starch, as boron deficiency could lead to impaired pollen viability (Sze *et al.*, 2006; Fang *et al.*, 2016). A formin-like protein 5 (*C.cajan_32517*) has also been identified and is known to be involved in pollen–pistil interaction (Boavida *et al.*, 2005). The expression and putative function of all these genes strongly suggests that they are associated to pollen viability and fertilization, potentially significant for seed setting directly or indirectly. These candidate genes would be a putative targets for functional validation and further study.

In pigeonpea, a hybrid breeding system based on cytoplasmic male sterility for A_4_ cytoplasm is well-established and commercialized (Saxena *et al.*, 2010). This provided an opportunity to validate the expression of genes involved in pollen fertility in two sets of male sterile genotypes and its fertile counterparts. Using qPCR, expression of genes encoding pectinesterase inhibitor 13 (*C.cajan_46391*), probable pectate lyase 3 (*C.cajan_44741*), serine/threonine-protein kinase (*C.cajan_07067*), sucrose-proton symporter 2 (*C.cajan_35390*) and an uncharacterized protein (*C.cajan_27282*) have been shown to have important roles in development of pollen. Further, the sequence analysis of the promoter regions of these genes has suggested their stimulus-dependent expression in response to light and phytohormones such as abscisic acid, auxin, salicylic acid, and methyl jasmonic acid. In addition, the preferential splicing events in seven of the genes exclusively in the floral tissues including bud, flower, stamen, pistil, sepal, and petal have suggested their critical role in normal pollen and seed development. Additionally, gene clusters represent interconnected and highly correlated genes that would be helpful in interpreting the biological role of those that are novel or uncharacterized. That is, clustering and visualization of the co-expressed gene network allows understanding of the basic function of genes that were annotated or unannotated genes forming a module in performing a specific function (Childs *et al.*, 2011). In this study, two uncharacterized proteins, *C.cajan_27282* and *C.cajan_28171*, have been shown to be involved in normal pollen development.

Indeed, growth and development have been understood as tightly regulated processes, through a multi-level regulation of gene expression. At the DNA level, chemical modifications of DNA and histone modification have been recognized as important regulatory mechanisms for controlling gene expression (Pfluger and Wagner, 2007; Saletore *et al.*, 2012). Such modifications have also been found at the RNA level, especially modifications of the mRNA, which have come to be known as the ‘epitranscriptome’. This is presumed to be an additional layer of regulation between DNA modification and post-translational modification. As DNA and histone modifications affect regulation of gene expression, mRNA modifications have recently been found to be crucial for proper plant development (Bodi *et al.*, 2012; Fray and Simpson, 2015). In mRNA, methylation of adenosine at the N^6^ position seems to be the most prevalent and has been found to be absolutely necessary for plant survival (Zhong *et al.*, 2008). This post-transcriptional modification is reversible, with so-called writer, reader, and eraser proteins (Fu *et al.*, 2014). Epitranscriptomics was explored in pigeonpea by identifying orthologous genes and looking at their expression patterns in pigeonpea tissues, previously reported in Arabidopsis (Zhong *et al.*, 2008). It was revealed that eight genes potentially encoding orthologous proteins were transcriptionally active in the CcGEA dataset. Moreover, some tissues related to active developmental processes such as embryo and immature/mature seed displayed higher abundance of these genes, suggesting that post-transcriptional regulation plays a crucial role in seed and embryo development in pigeonpea. These genes could be studied further for their involvement in modulating important agronomical traits related to seed development and germination.

A gene expression atlas has been developed in different legumes such as *Medicago*, *Lotus*, soybean, pea, black-eyed pea, and peanut with a focus on important traits. For instance, in the case of *Medicago* and pea, genes preferentially expressed in nodules have been emphasized, whereas in the case of the peanut, genes involved in geocarpy have also been described. Similarly, in the case of soybean and black-eyed pea, seed development and maturation have been focused, respectively. In pigeonpea, the CcGEA has facilitated identification of candidate genes of agronomic importance for possible deployment of GAB. This has been illustrated with the identification of candidate genes associated with pollen fertility and fertilization crucial for seed formation, providing potential candidates for future studies. Likewise, the CcGEA provides a compendium of genes identified in 30 diverse tissues that are clustered based on their expression patterns. Gene clusters have been identified with aerial and underground preferential expression, in addition to those vital for floral morphology and symmetry. These clusters could be further analysed to identify candidate genes for different agronomic traits. For instance, Cl-I has been shown to be enriched with stress-responsive genes, especially for drought and heat stress. These included genes encoding stress-induced protein (SAM22), universal stress protein-A, heat shock proteins, protein EARLY RESPONSIVE TO DEHYDRATION 15 (ERD15), and many stress-related proteins (SRP). This cluster could be analysed for identifying candidate genes that would be crucial for bolstering hardiness to the crop. Furthermore, this resource could also be utilized to look into the baseline expression of genes studied in other legumes/crops in different tissues of pigeonpea that could be traced at different developmental stages. Thus, this resource could be valuable for the scientific community not only working in pigeonpea but also in related legume crops.

## Conclusions

The gene expression atlas (CcGEA) developed in pigeonpea complements the genome sequence of pigeonpea and other genomic resources in understanding gene functions and their biological role. The CcGEA represents a comprehensive dataset of genes expressed in 30 diverse tissues across five developmental stages from embryo to senescence. The dataset has been analysed using pairwise comparison, clustering and correlation network analysis. The efficacy of the CcGEA has been demonstrated by identifying a gene network of 28 genes putatively regulated by a pollen-specific SF3 and a sucrose–proton symporter. Gene expression studies using two sets of male sterile and fertile genotypes revealed 20 genes crucial for pollen development. The role of these genes could also be ascertained in floral tissues with exclusive splicing variants identified in these tissues. This study also provide genes that would be excellent candidates for a reverse genetics approach to determine their roles in pollen fertility and seed formation. Likewise, this dataset could be further analysed to identify candidate genes for various agronomic traits such as abiotic stress tolerance, especially for drought and heat stress. The CcGEA would also be useful in looking at the basal expression of genes when investigating mutant genotypes or any candidate gene expression for a specific agronomic trait. This resource will be valuable for studying the genes expressed in specialized tissues or organ systems such as nodules, flowers and pods in pigeonpea or related legumes. With further refinement of the existing draft genome assembly or the development of a pan genome, the CcGEA could be improved further and in that scenario, it will provide more and comprehensive insights into gene expression.

## Supplementary Material

Supplementary Data

## References

[CIT0001] Alves-Carvalho S Aubert G Carrère S 2015 Full-length *de novo* assembly of RNA-seq data in pea (*Pisum sativum* L.) provides a gene expression atlas and gives insights into root nodulation in this species. The Plant Journal 84, 1–19. 26296678 10.1111/tpj.12967

[CIT0002] Baltz R Domon C Pillay DT Steinmetz A 1992a Characterization of a pollen-specific cDNA from sunflower encoding a zinc finger protein. The Plant Journal 2, 713–721. 1302629

[CIT0003] Baltz R Evrard JL Domon C Steinmetz A 1992b A LIM motif is present in a pollen-specific protein. The Plant Cell 4, 1465–1466. 1467648 10.1105/tpc.4.12.1465 PMC160233

[CIT0004] Benedito VA Torres-Jerez I Murray JD 2008 A gene expression atlas of the model legume *Medicago truncatula*. The Plant Journal 55, 504–513. 18410479 10.1111/j.1365-313X.2008.03519.x

[CIT0005] Boavida LC Vieira AM Becker JD Feijó JA 2005 Gametophyte interaction and sexual reproduction: how plants make a zygote. The International Journal of Developmental Biology 49, 615–632. 16096969 10.1387/ijdb.052023lb

[CIT0006] Bodi Z Zhong S Mehra S Song J Graham N Li H May S Fray RG 2012 Adenosine methylation in Arabidopsis mRNA is associated with the 3’ end and reduced levels cause developmental defects. Frontiers in Plant Science 3, 48. 22639649 10.3389/fpls.2012.00048 PMC3355605

[CIT0007] Childs KL Davidson RM Buell CR 2011 Gene coexpression network analysis as a source of functional annotation for rice genes. PLoS One 6, e22196. 21799793 10.1371/journal.pone.0022196 PMC3142134

[CIT0008] Clevenger J Chu Y Scheffler B Ozias-Akins P 2016 A developmental transcriptome map for allotetraploid *Arachis hypogaea*. Frontiers in Plant Science 7, 1446. 27746793 10.3389/fpls.2016.01446 PMC5043296

[CIT0009] Conesa A Götz S García-Gómez JM Terol J Talón M Robles M 2005 Blast2GO: a universal tool for annotation, visualization and analysis in functional genomics research. Bioinformatics 21, 3674–3676. 16081474 10.1093/bioinformatics/bti610

[CIT0010] Egea‐Cortines M Saedler H Sommer H 1999 Ternary complex formation between the MADS‐box proteins SQUAMOSA, DEFICIENS and GLOBOSA is involved in the control of floral architecture in *Antirrhinum majus*. The EMBO Journal 18, 5370–5379. 10508169 10.1093/emboj/18.19.5370 PMC1171606

[CIT0011] Everitt BS Hothorn TA 2005 Handbook of Statistical Analyses Using R. Boca Raton: CRC Press, Ch. 15.

[CIT0012] Fang K Zhang W Xing Y Zhang Q Yang L Cao Q Qin L 2016 Boron toxicity causes multiple effects on *Malus domestica* pollen tube growth. Frontiers in Plant Science 7, 208. 26955377 10.3389/fpls.2016.00208 PMC4768074

[CIT0013] Fray RG Simpson GG 2015 The Arabidopsis epitranscriptome. Current Opinion in Plant Biology 27, 17–21. 26048078 10.1016/j.pbi.2015.05.015

[CIT0014] Fu Y Dominissini D Rechavi G He C 2014 Gene expression regulation mediated through reversible m⁶A RNA methylation. Nature Reviews. Genetics 15, 293–306. 10.1038/nrg3724 24662220

[CIT0015] Gatto A Torroja-Fungairiño C Mazzarotto F Cook SA Barton PJ Sánchez-Cabo F Lara-Pezzi E 2014 FineSplice, enhanced splice junction detection and quantification: a novel pipeline based on the assessment of diverse RNA-Seq alignment solutions. Nucleic Acids Research 42, e71. 24574529 10.1093/nar/gku166 PMC4005686

[CIT0016] Hasty J McMillen D Isaacs F Collins JJ 2001 Computational studies of gene regulatory networks: in numero molecular biology. Nature Reviews. Genetics 2, 268–279. 10.1038/35066056 11283699

[CIT0017] Hollender CA Kang C Darwish O Geretz A Matthews BF Slovin J Alkharouf N Liu Z 2014 Floral transcriptomes in woodland strawberry uncover developing receptacle and anther gene networks. Plant Physiology 165, 1062–1075. 24828307 10.1104/pp.114.237529 PMC4081322

[CIT0018] Holter NS Maritan A Cieplak M Fedoroff NV Banavar JR 2001 Dynamic modeling of gene expression data. Proceedings of the National Academy of Sciences, USA 98, 1693–1698. 10.1073/pnas.98.4.1693 PMC29319 11172013

[CIT0019] Howe E Holton K Nair S Schlauch D Sinha R Quackenbush J 2010 Mev: multiexperiment viewer. In Biomedical informatics for cancer research. New York: Springer US, 267–277.

[CIT0020] Katta MA Khan AW Doddamani D Thudi M Varshney RK 2015 NGS-QCbox and raspberry for parallel, automated and rapid quality control analysis of large-scale next generation sequencing (Illumina) data. PLoS One 10, e0139868. 26460497 10.1371/journal.pone.0139868 PMC4604202

[CIT0021] Katz Y Wang ET Silterra J Schwartz S Wong B Thorvaldsdóttir H Robinson JT Mesirov JP Airoldi EM Burge CB 2015 Quantitative visualization of alternative exon expression from RNA-seq data. Bioinformatics 31, 2400–2402. 25617416 10.1093/bioinformatics/btv034 PMC4542614

[CIT0022] Kim D Pertea G Trapnell C Pimentel H Kelley R Salzberg SL 2013 TopHat2: accurate alignment of transcriptomes in the presence of insertions, deletions and gene fusions. Genome Biology 14, R36. 23618408 10.1186/gb-2013-14-4-r36 PMC4053844

[CIT0023] Kole C Muthamilarasan M Henry R 2015 Application of genomics-assisted breeding for generation of climate resilient crops: progress and prospects. Frontiers in Plant Science 6, 563. 26322050 10.3389/fpls.2015.00563 PMC4531421

[CIT0024] Langfelder P Horvath S 2007 Eigengene networks for studying the relationships between co-expression modules. BMC Systems Biology 1, 54. 18031580 10.1186/1752-0509-1-54 PMC2267703

[CIT0025] Langfelder P Horvath S 2008 WGCNA: an R package for weighted correlation network analysis. BMC Bioinformatics 9, 559. 19114008 10.1186/1471-2105-9-559 PMC2631488

[CIT0026] Lemoine R Bürkle L Barker L Sakr S Kühn C Regnacq M Gaillard C Delrot S Frommer WB 1999 Identification of a pollen-specific sucrose transporter-like protein NtSUT3 from tobacco. FEBS Letters 454, 325–330. 10431832 10.1016/s0014-5793(99)00843-1

[CIT0027] Lescot M Déhais P Thijs G Marchal K Moreau Y Van de Peer Y Rouzé P Rombauts S 2002 PlantCARE, a database of plant cis-acting regulatory elements and a portal to tools for in silico analysis of promoter sequences. Nucleic Acids Research 30, 325–327. 11752327 10.1093/nar/30.1.325 PMC99092

[CIT0028] Libault M Farmer A Joshi T Takahashi K Langley RJ Franklin LD He J Xu D May G Stacey G 2010 An integrated transcriptome atlas of the crop model *Glycine max*, and its use in comparative analyses in plants. The Plant Journal 63, 86–99. 20408999 10.1111/j.1365-313X.2010.04222.x

[CIT0029] Livak KJ Schmittgen TD 2001 Analysis of relative gene expression data using real-time quantitative PCR and the 2(–ΔΔ*C*_T_) method. Methods 25, 402–408. 11846609 10.1006/meth.2001.1262

[CIT0030] Lohmann JU Weigel D 2002 Building beauty: the genetic control of floral patterning. Developmental Cell 2, 135–142. 11832239 10.1016/s1534-5807(02)00122-3

[CIT0031] Lopes CT Franz M Kazi F Donaldson SL Morris Q Bader GD 2010 Cytoscape web: an interactive web-based network browser. Bioinformatics 26, 2347–2348. 20656902 10.1093/bioinformatics/btq430 PMC2935447

[CIT0032] Ma J Wei H Song M Pang C Liu J Wang L Zhang J Fan S Yu S 2012 Transcriptome profiling analysis reveals that flavonoid and ascorbate-glutathione cycle are important during anther development in upland cotton. PLoS One 7, e49244. 23155472 10.1371/journal.pone.0049244 PMC3498337

[CIT0033] Masuko H Endo M Saito H 2006 Anther-specific genes, which expressed through microsporogenesis, are temporally and spatially regulated in model legume, *Lotus japonicus*. Genes & Genetic Systems 81, 57–62. 16607042 10.1266/ggs.81.57

[CIT0034] Mutwil M Usadel B Schütte M Loraine A Ebenhöh O Persson S 2010 Assembly of an interactive correlation network for the Arabidopsis genome using a novel heuristic clustering algorithm. Plant Physiology 152, 29–43. 19889879 10.1104/pp.109.145318 PMC2799344

[CIT0035] O’Rourke JA Iniguez LP Fu F 2014 An RNA-Seq based gene expression atlas of the common bean. BMC Genomics 15, 866. 25283805 10.1186/1471-2164-15-866 PMC4195886

[CIT0036] Pazhamala L Saxena RK Singh VK 2015 Genomics-assisted breeding for boosting crop improvement in pigeonpea (*Cajanus cajan*). Frontiers in Plant Science 6, 50. 25741349 10.3389/fpls.2015.00050 PMC4330709

[CIT0037] Pazhamala LT Agarwal G Bajaj P Kumar V Kulshreshtha A Saxena RK Varshney RK 2016 Deciphering transcriptional programming during pod and seed development using RNA-Seq in pigeonpea (*Cajanus cajan*). PLoS One 11, e0164959. 27760186 10.1371/journal.pone.0164959 PMC5070767

[CIT0038] Pfluger J Wagner D 2007 Histone modifications and dynamic regulation of genome accessibility in plants. Current Opinion in Plant Biology 10, 645–652. 17884714 10.1016/j.pbi.2007.07.013 PMC2140274

[CIT0039] Ribeiro A Akkermans AD van Kammen A Bisseling T Pawlowski K 1995 A nodule-specific gene encoding a subtilisin-like protease is expressed in early stages of actinorhizal nodule development. The Plant Cell 7, 785–794. 7647567 10.1105/tpc.7.6.785 PMC160833

[CIT0040] Robinson JT Thorvaldsdóttir H Winckler W Guttman M Lander ES Getz G Mesirov JP 2011 Integrative genomics viewer. Nature Biotechnology 29, 24–26. 10.1038/nbt.1754 PMC3346182 21221095

[CIT0041] Sakuraba Y Jeong J Kang MY Kim J Paek NC Choi G 2014 Phytochrome-interacting transcription factors PIF4 and PIF5 induce leaf senescence in Arabidopsis. Nature Communications 5, 4636. 10.1038/ncomms5636 25119965

[CIT0042] Saletore Y Meyer K Korlach J Vilfan ID Jaffrey S Mason CE 2012 The birth of the Epitranscriptome: deciphering the function of RNA modifications. Genome Biology 13, 175. 23113984 10.1186/gb-2012-13-10-175 PMC3491402

[CIT0043] Sauer N 2007 Molecular physiology of higher plant sucrose transporters. FEBS Letters 581, 2309–2317. 17434165 10.1016/j.febslet.2007.03.048

[CIT0044] Saxena KB Sultana R Saxena RK Kumar RV Sandhu JS Rathore A Kishor PB Varshney RK 2011 Genetics of fertility restoration in A_4_-based, diverse maturing hybrids of pigeonpea [*Cajanus cajan* (L.) Millsp.]. Crop Science 51, 574–578.

[CIT0045] Saxena KB Sultana R Mallikarjuna N Saxena RK Kumar RV Sawargaonkar SL Varshney RK 2010 Male‐sterility systems in pigeonpea and their role in enhancing yield. Plant Breeding 129, 125–134.

[CIT0046] Severin AJ Woody JL Bolon YT 2010 RNA-Seq atlas of *Glycine max*: a guide to the soybean transcriptome. BMC Plant Biology 10, 160. 20687943 10.1186/1471-2229-10-160 PMC3017786

[CIT0047] Shen-Orr SS Milo R Mangan S Alon U 2002 Network motifs in the transcriptional regulation network of *Escherichia coli*. Nature Genetics 31, 64–68. 11967538 10.1038/ng881

[CIT0048] Singh VK Khan AW Saxena RK 2016 Next-generation sequencing for identification of candidate genes for Fusarium wilt and sterility mosaic disease in pigeonpea (*Cajanus cajan*). Plant Biotechnology Journal 14, 1183–1194. 26397045 10.1111/pbi.12470 PMC5054876

[CIT0049] Sinha P Singh VK Suryanarayana V Krishnamurthy L Saxena RK Varshney RK 2015 Evaluation and validation of housekeeping genes as reference for gene expression studies in pigeonpea (*Cajanus cajan*) under drought stress conditions. PLoS One 10, e0122847. 25849964 10.1371/journal.pone.0122847 PMC4388706

[CIT0050] Somerville CR Ogren WL 1980 Inhibition of photosynthesis in *Arabidopsis* mutants lacking leaf glutamate synthase activity. Nature 286, 257–259.

[CIT0051] Szczyglowski K Hamburger D Kapranov P de Bruijn FJ 1997 Construction of a *Lotus japonicus* late nodulin expressed sequence tag library and identification of novel nodule-specific genes. Plant Physiology 114, 1335–1346. 9276951 10.1104/pp.114.4.1335 PMC158426

[CIT0052] Sze H Frietsch S Li X Bock KW Harper JF 2006 Genomic and molecular analyses of transporters in the male gametophyte. In: The pollen tube. Berlin, Heidelberg: Springer, 71–93.

[CIT0053] Todd J Post-Beittenmiller D Jaworski JG 1999 KCS1 encodes a fatty acid elongase 3-ketoacyl-CoA synthase affecting wax biosynthesis in *Arabidopsis thaliana*. The Plant Journal 17, 119–130. 10074711 10.1046/j.1365-313x.1999.00352.x

[CIT0054] Trapnell C Hendrickson DG Sauvageau M Goff L Rinn JL Pachter L 2012b Differential analysis of gene regulation at transcript resolution with RNA-seq. Nature Biotechnology 31, 46–53. 10.1038/nbt.2450 PMC3869392 23222703

[CIT0055] Trapnell C Roberts A Goff L Pertea G Kim D Kelley DR Pimentel H Salzberg SL Rinn JL Pachter L 2012a Differential gene and transcript expression analysis of RNA-seq experiments with TopHat and Cufflinks. Nature Protocols 7, 562–578. 22383036 10.1038/nprot.2012.016 PMC3334321

[CIT0056] Trapnell C Williams BA Pertea G Mortazavi A Kwan G van Baren MJ Salzberg SL Wold BJ Pachter L 2010 Transcript assembly and quantification by RNA-Seq reveals unannotated transcripts and isoform switching during cell differentiation. Nature Biotechnology 28, 511–515. 10.1038/nbt.1621 PMC3146043 20436464

[CIT0057] Varshney RK Chen W Li Y 2012 Draft genome sequence of pigeonpea (*Cajanus cajan*), an orphan legume crop of resource-poor farmers. Nature Biotechnology 30, 83–89. 10.1038/nbt.2022 22057054

[CIT0058] Varshney RK Mohan SM Gaur PM 2013 Achievements and prospects of genomics-assisted breeding in three legume crops of the semi-arid tropics. Biotechnology Advances 31, 1120–1134. 23313999 10.1016/j.biotechadv.2013.01.001

[CIT0059] Verdier J Torres-Jerez I Wang M Andriankaja A Allen SN He J Tang Y Murray JD Udvardi MK 2013 Establishment of the *Lotus japonicus* gene expression atlas (LjGEA) and its use to explore legume seed maturation. The Plant Journal 74, 351–362. 23452239 10.1111/tpj.12119

[CIT0060] Yao S Jiang C Huang Z Torres‐Jerez I Chang J Zhang H Udvardi M Liu R Verdier J 2016 The *Vigna unguiculata* gene expression atlas (VuGEA) from *de novo* assembly and quantification of RNA‐seq data provides insights into seed maturation mechanisms. The Plant Journal 88, 318–327. 10.1111/tpj.13279 27448251

[CIT0061] Zhong S Li H Bodi Z Button J Vespa L Herzog M Fray RG 2008 MTA is an Arabidopsis messenger RNA adenosine methylase and interacts with a homolog of a sex-specific splicing factor. The Plant Cell 20, 1278–1288. 18505803 10.1105/tpc.108.058883 PMC2438467

