# Supplementary material for: Gene expression atlas of pigeonpea and its application to gain insights into genes associated with pollen fertility implicated in seed formation
Source: J Exp Bot. 2017 Feb 23;68(8):2037–54. doi: 10.1093/jxb/erx010 (PMC5429002; doi:10.1093/jxb/erx010)
Supplement: Supplementary Data [file erx010_Supplementary_Data.zip › supplementary_figures_S1_S2.pdf]

Gene expression atlas of pigeonpea and its application to gain insights on genes associated with pollen fertility implicated in seed formation

Lekha T Pazhamala, Shilp Purohit, Rachit K Saxena, Vanika Garg, Laxmanan Krishnamurthy, Jerome Verdier, and Rajeev K Varshney

Supplementary Figures

Fig. S1: Three experimental setup of pigeonpea grown under glasshouse conditions for sampling different tissues. For harvesting tissues from the initial germinal stages, seeds were germinated in petri plates, while for the seedling stages, seedlings were grown in paper cups containing sterile sand. Medium sized pots containing vertisol-sand mix were used to grow plants for harvesting tissues from vegetative reproductive and senescence stages.

Fig. S2: k-means clustering of 28,793 significantly expressed genes into ten clusters

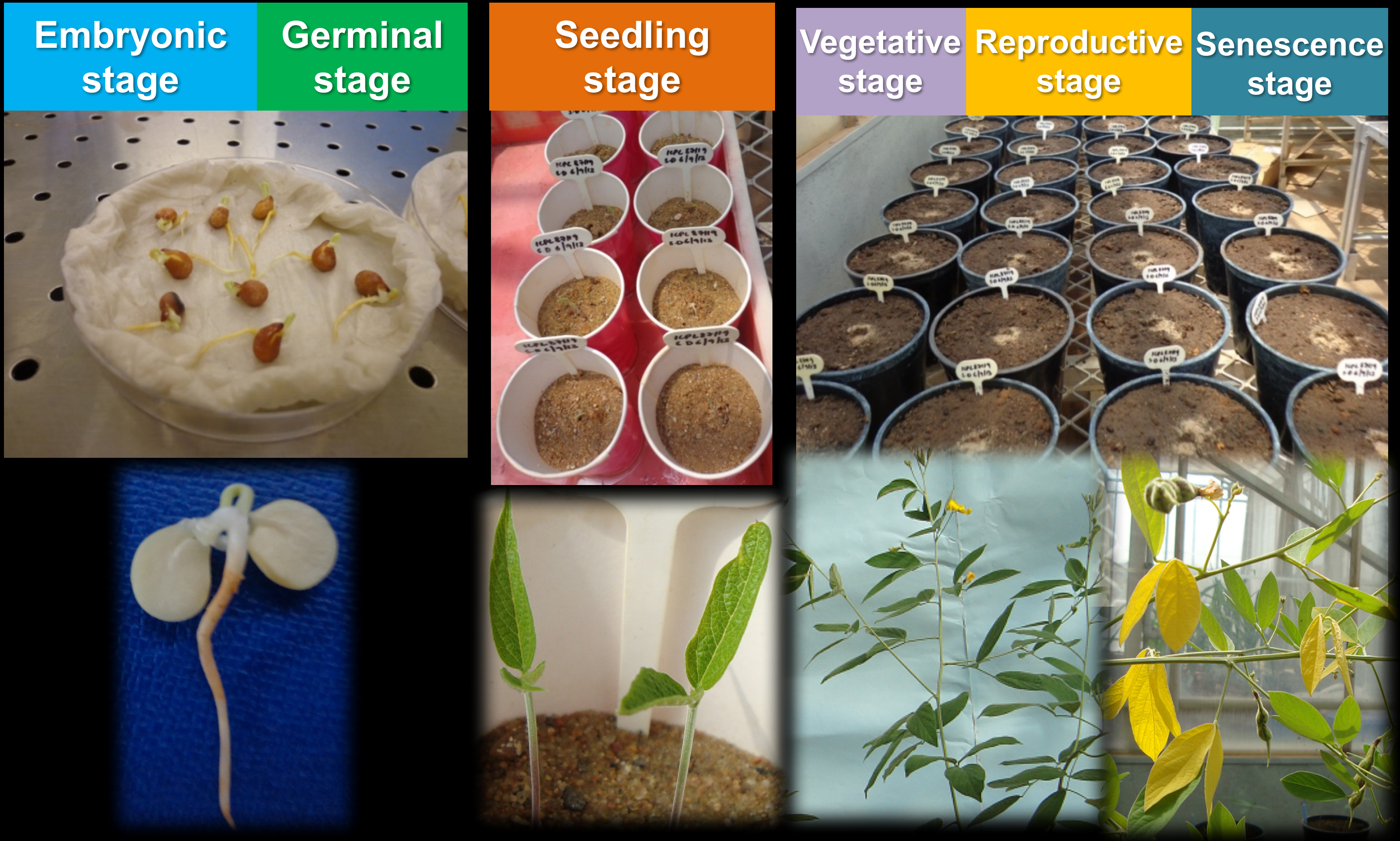
Embryonic stage
Germinal stage
Seedling stage
Vegetative stage
Reproductive stage
Senescence stage

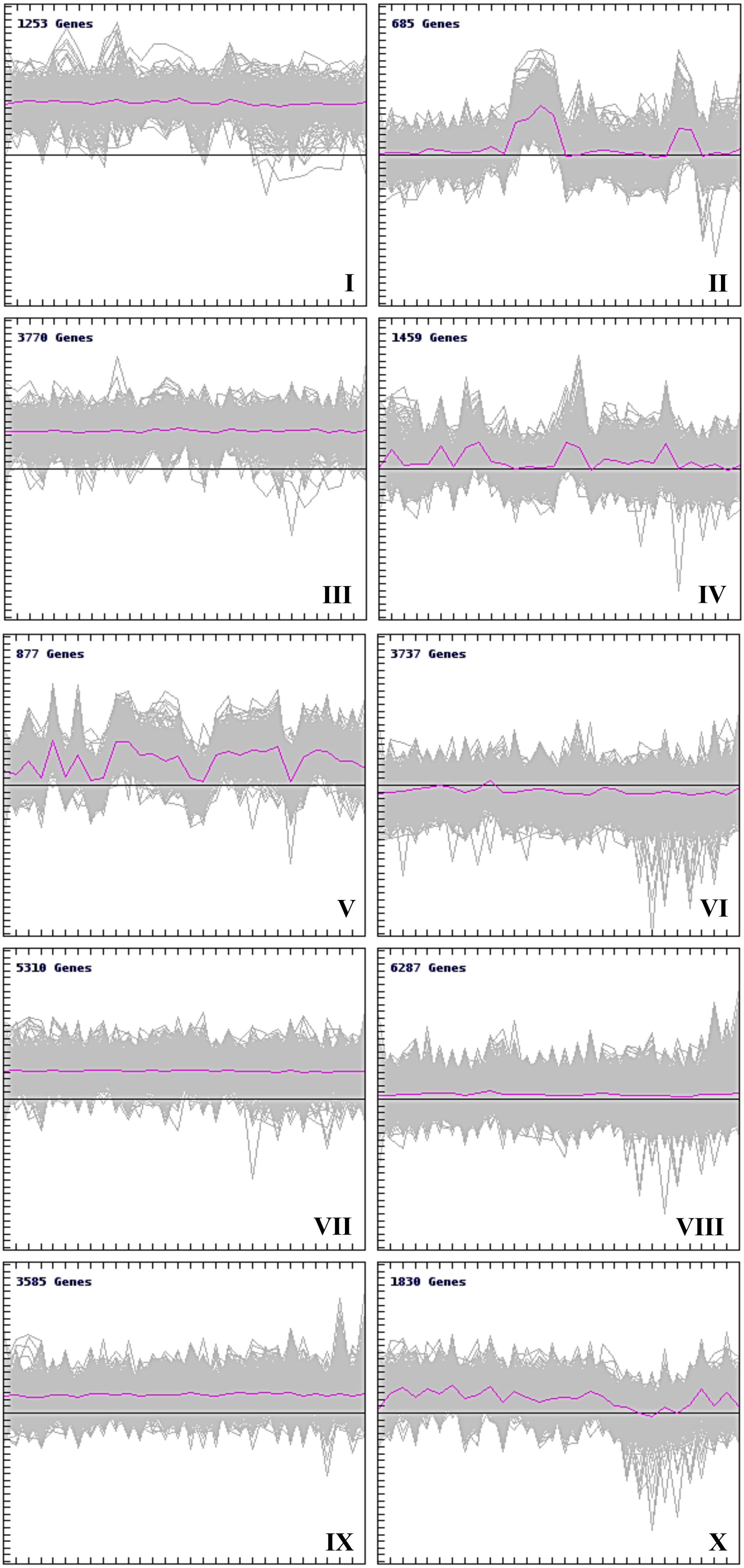
1253 Genes
I
685 Genes
II
3770 Genes
III
1459 Genes
IV
877 Genes
V
3737 Genes
VI
5310 Genes
VII
6287 Genes
VIII
3585 Genes
IX
1830 Genes
X
